# *Bombyx mori* Transcription Factors: Genome-Wide Identification, Expression Profiles and Response to Pathogens by Microarray Analysis

**DOI:** 10.1673/031.012.4001

**Published:** 2012-03-24

**Authors:** Lulin Huang, Tingcai Cheng, Pingzhen Xu, Ting Fang, Qingyou Xia

**Affiliations:** ^1^ State Key Laboratory of Silkworm Genome Biology, Southwest University, Chongqing 400716, China

**Keywords:** bacterial injection, developmental stages, multiple tissues, oral infection, pathogens, sexual dimorphism, silkworm, transcription factors

## Abstract

Transcription factors are present in all living organisms, and play vital roles in a wide range of biological processes. Studies of transcription factors will help reveal the complex regulation mechanism of organisms. So far, hundreds of domains have been identified that show transcription factor activity. Here, 281 reported transcription factor domains were used as seeds to search the transcription factors in genomes of *Bombyx mori* L. (Lepidoptera: Bombycidae) and four other model insects. Overall, 666 transcription factors including 36 basal factors and 630 other factors were identified in *B. mori* genome, which accounted for 4.56% of its genome. The silkworm transcription factors' expression profiles were investigated in relation to multiple tissues, developmental stages, sexual dimorphism, and responses to oral infection by pathogens and direct bacterial injection. These all provided rich clues for revealing the transcriptional regulation mechanism of silkworm organ differentiation, growth and development, sexual dimorphism, and response to pathogen infection.

## Introduction

Gene expression and regulation is a core component of functional genomics research. One of the most vital reasons for phenotypic differences in organisms is regulation of gene expression achieved by a network of transcription factors and their target genes. DNA transcription factors combining the cisacting elements and activating the downstream target genes are the key steps in gene expression. Transcription factors include basic factors, activators, coactivators, and some regulators. They are present in all living organisms, and play a vital role in a wide range of biological processes such as growth, development, stress responses, and other physiological processes (Saibo et al. 2009).

So far, hundreds of domain families showing transcription factor activity have been identified, including C2H2-type zinc finger, helix-loop-helix DNA-binding (HLH), homeobox, Myb-like DNA-binding and GATA. The C2H2-type zinc finger proteins belong to the largest family of regulatory transcription factors, playing important roles in growth and development in animals and also in plant systems (Hsu et al. 2006). Similarly, the HLH and homeobox families are widespread in the biosphere. Some transcription factors such as the MYB superfamily mainly exist in plants, while other families such as P53 proteins are mainly distributed in higher animals (Riechmann et al. 2000).

In the last decade, with the rapid development of genomics, genome-wide screening of transcription factors in some model species has been performed to give a better understanding of their distribution in species. For example, Riechmann et al. (2000) identified 1533 transcription factors in *Arabidopsis*, which represented 5.9% its genome. Some researchers are dedicated to the collection of transcription factors and establishment of databases; for instance, the DNA-binding domain (DBD) databases provide a collection place for predicted sequence-specific DNA-binding transcription factors for all publicly available proteomes (Wilson et al. 2008). Guo et al. (2008) developed a comprehensive plant transcription factor database PlantTFDB (http://planttfdb.cbi.pku.edu.cn), which contains 26,402 transcription factors predicted from 22 plant species in 2008. These publications suggest that transcription factors have attracted wide interest from researchers.

The silkworm *Bombyx mori* L. (Lepidoptera: Bombycidae), with its cocoon-spinning habit, is a representative species of Lepidoptera and is considered an important model organism to investigate biological phenomena including of development, gene regulation, and morphological innovation. In the present study, to achieve better understanding of gene regulation mechanism in *B. mori*, the transcription factors in the silkworm genome were investigated. Transcription factors in the model insects *Drosophila melanogaster, Anopheles gambiae, Tribolium castaneum*, and *Apis mellifera* were also investigated. The expression profiles of silkworm transcription factors were also analyzed in multiple tissues and at five developmental stages and according to sexual dimorphism using microarray data. Finally, responses to oral infection by pathogens and direct bacterial injection were also analyzed, in the expectation of providing clues to understanding silkworm transcriptional regulation mechanisms following pathogen infection.

## Materials and Methods

### Sanger protein family (Pfam) domains prediction of insect proteomes

The proteomes used in this report were downloaded from the following websites: the predicted *B. mori* proteome was downloaded from the silkDB (http://silkworm.swu.edu.cn/silkdb/, 9 x) (Duan et al. 2009); the predicted proteomes of *D. melanogaster* (Release 4.3) were obtained from FlyBase (www.flybase.org); the predicted proteomes of *A. gambiae* (release 3.6) downloaded from UCSC Genome Browser Database (www.genome.ucsc.edu); the predicted proteomes of *A. mellifera* (Amel_2.0) and *T. castaneum* (Tcas_1.0) were taken from HGSC (www.hgsc.bcm.tmc.edu); Pfam domains were predicted online using the above proteomes in the Sanger Pfam database (http://pfam.sanger.ac.uk/search/batch).

### Transcription factors, Pfam domain seeds and transcription factor identification

Transcription initiation requires the transcription factors to act in a defined order to build a complex that is joined by RNA polymerase (Mermelstein et al. 1989). To determine the basal transcription factors in *B. mori*, the Sanger Pfam database was searched to find domains identified with RNA polymerase II transcription factor activity or transcription initiation factor activity. Because the sigma-factor controls promoter recognition, the sigma-factor domains for basal transcription factor domains (Borukhov and Severinov 2002) were also searched. In total, 51 domains were identified as basal transcription factor domains; see Table 1 for the list of identified domains. Other transcription factors included transcription activators, co-activators, and mediators. The Pfam database showed 230 domains identified as other transcription factors; see Table 2 for the list of identified domains. Using these domains as seeds, the silkworm proteome and the other four insect proteome domains were searched. Using the criterion of domain e-value < 1e—5, genes containing at least one identified domain as transcription factors were selected.

### Construction of phylogenetic tree of Myb/SANT-like family

The predicted amino acid sequences of the Myb/SANT-like family domains were obtained using Pfam search tools. The amino acid sequences were initially aligned using ClustalW and then adjusted manually. To construct the phylogenetic trees with the obtained Myb/SANT-like domain sequences, neighbor-joining trees were prepared (100 bootstrap) using MEGA 4.0.

### Multiple tissues expression microarray data

The microarray data of multiple tissues expression of day three of the fifth instar were downloaded from the silkworm genome database (http://www.silkdb.org/microarray/download.html) (10,393 active transcripts) (Xia et al. 2007). The expression data of each gene in each tissue were averaged from 4–6 repeats. For each gene, if the averaged expression signal was > 400, it was considered to have expression. Only genes that showed expression in one tissue or > 10 times the signal units of other tissues were considered as tissue-specific genes.

### Silkworm sample preparation for microarray experiment

***Developmental stages*.** The Chinese silkworm strain *Dazao* was reared at a stable temperature of 25 °C. We surveyed gene expression of males and females, respectively, at the stages of mature larva, during spinning (12, 24, 36, 48, 60, and 72 hours of spinning), pupa (1, 2, 3, 4, 5, and 6 days), and moth (1 day). The whole bodies of three silkworms were obtained in each sample.

***Oral infection by microbes*.** The Chinese silkworm strain *Dazao* was reared at a stable temperature of 25 °C. The larvae stopped feeding on day three of the fifth instar for the infection experiments. Microbes BmNPV, *Bacillus bombyseptieus* and *Beauveria bassinana* and *Escherichia coli* (DH5α) were used for oral infection. Bacterium oral infection used our previous reported method (Huang et al. 2009). BmNPV was separated and purified from *B. mori* larvae. The *B. bassinana* spores were cultured in potato solid medium. About 10^5^ BmNPV and 10^6^ *B. bassinana* spores for each larva were thoroughly mixed with food and orally infected for three hours and then raised at 25 °C with approximately 70% RH. After that, time calculation commenced. The whole bodies of three larvae were collected as one sample at different time intervals after infection.

***Microbe injection*.** Preparation of bacterial *B. thuringiensis and E. coli* injection samples for microarray analysis used methods from Huang et al. (2009).

### mRNA extraction and microarray hybridization

***RNA extraction*.** Obtained samples were frozen in liquid nitrogen immediately after material collection. Three independent samples were obtained. After homogenization of the larvae in liquid nitrogen, the resulting powders were added to 2.0 mL centrifuge tubes (each containing approximately 0.1 g). TRIzol reagent (Invitrogen) was then added and total RNA was extracted according to the manufacturer's instructions. The total RNA templates were quantified by spectrophotometry and subjected to 1.0% formaldehyde denatured agarose gel electrophoresis. Then, samples were precipitated in 100% ethanol and sent to CapitalBio Corporation for microarray analysis or stored at -80 °C for further analysis.

***Microarray hybridisation and original data normalization*.** Gene expression analysis was performed using the Affymetrix Silkworm GeneChip kit (www.affymetrix.com) according to instructions in the Affymetrix GeneChip expression manual. The microarray hybridisation and data normalisation analyses were performed by CapitalBio Corporation (Xia et al. 2007). Procedures were performed as described in detail on the CapitalBio website (www.capitalbio.com). Briefly, total RNA was purified using NucleoSpin® RNA clean-up kit (Macherey-Nagel, www.mnnet.com). Then, formaldehyde denaturing gel electrophoresis was used to detect the RNA quality.

A dual-dye experiment was used to analyze the expression patterns. For developmental stages, one of the dyes (Cy3) was used to label a female sample, while the other dye labeled a male sample. For microbe infection, Cy3 was used to label a control sample, while the other dye labeled an induced sample.

### Data analysis

For each gene, if the averaged expression signal was > 400, it was considered to have expression. Only genes that showed expression in one tissue or one developmental stage with > 10 times more than others were considered as tissue- or developmental stage-specific genes. Genes with expression signal units more than twice as high in either the male or female were considered as sexual differential genes. For microbe infection, the induced ratios comparing to non-induced more than twofold were considered as induced genes.

## Results

### Transcription factors in *B. mori*

**Figure 1.**
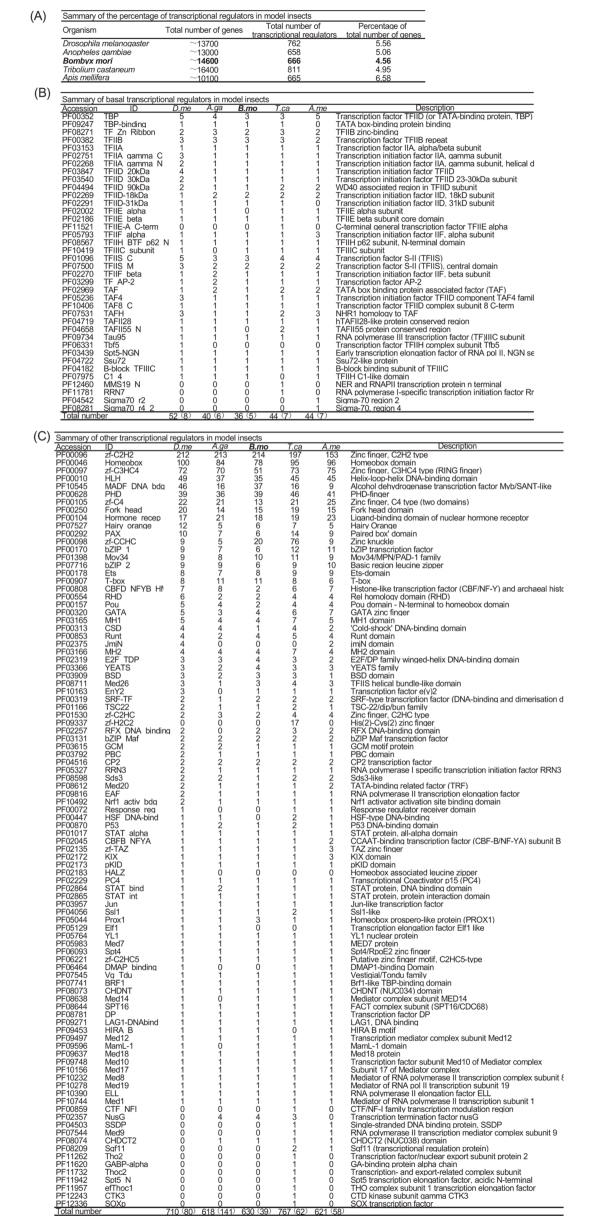
Content of transcription factors in insects genomes. (a) Summary of the percentage of transcriptional factors in model insects (*Bombyx mori, Drosophila melanogaster, Anopheles gambiae, Tribolium castaneum*, and *Apis mellifera*). (b) Summary of basal factors in model insects. The brackets showed the gene numbers containing at least two types of basal domains. (c) Summary of other transcriptional factors in model insects. The brackets showed the gene numbers containing at least two types of other domains. The list of identified basal factors and other factors of *Bombyx mori* are shown in Table 3 and Table 4. The list of identified basal factors and other factors of *Drosophila melanogaster* are shown in Table 5 and Table 9. The list of identified basal factors and other factors of *Anopheles gambiae* are shown in Table 6 and Table 10. The list of identified basal factors and other factors of *Tribolium castaneum* are shown in Table 7 and Table 11. The list of identified basal factors and other factors of *Apis mellifera* are shown in Table 8 and Table 12. High quality figures are available online.

To determine the transcription factors in the *B. mori* genome, the Sanger Pfam database was searched to find domains with transcription factor activity. Among all domains in the Sanger Pfam database, 51 were identified as basal factors (basal domains) and 230 as other transcription factor domains. Tables 1 and 2 show the domain accession, domain ID and description of the individual identified basal and other transcription factors. After that, using these identified domains as searching seeds, we searched the Sanger Pfam predicted domains of the silkworm predicted proteome with e-value < e—5 to find genes corresponding to the silkworm transcription factors. Overall, in the silkworm genome, 666 transcription factors in total were determined, including 36 basal and 630 other transcription factors, which accounted for 4.56% of about 14,600 genes of the silkworm genome (Figure 1A).

### Basal transcription factors in *B. mori*

Generally, in eukaryotes, basal transcription factors are necessary for transcription to occur and thereby control expression of a wide range of genes. A search of the 51 identified basal domains showed 29 in the silkworm that were involved in 36 genes. Among them, five genes contained at least two types of basal domains (Figure 1B). Table 3 shows the gene ID, domain accession, e-value, domain ID, and description of the identified silkworm basal transcription factors. Among silkworm basal families, TBP, TFIIB, and TFIISC were the largest with three members each. The remaining families were smaller, including TFIIB zinc-binding, transcription initiation factor II D (18kD subunit), transcription initiation factor IIA, TATA box binding protein associated factor, transcription factor Tfb2, and transcription factor Tau95, all with only one or two genes each. Interestingly, of the 12 searched sigma-factor-related domains (e.g., sigma-54 factor and sigma-70 factor), none were identified in the silkworm genome.

### Other transcription factors in *B. mori*

Other transcription factors mainly include activators which can recognize specific short consensus-elements and bind to sites in the promoter or in enhancers such as the zinc finger motif, homeodomain DNA-binding domain, basic HLH (bHLH) domain, bZIP leucine zipper motif, and other DNA-binding domains or transcription regulators (Rachez and Freedman 2001). Searching the identified 230 other transcription factor domains showed 70 in total identified in the silkworm involved in 630 genes. Among them, 39 contained at least two types of other factor domains (Figure 1C). Table 4 shows the gene ID, accession, e-value, domain ID, and the description of the other identified silkworm transcription factors.

Like other species, the largest silkworm transcription factor family was also the zinc finger, including of the C2H2 type, C3HC4 type, PHD-finger, C4 type (two domains), zinc knuckle, GATA zinc finger, and other small families (Figure 1C). The C2H2-type zinc fingers contain a short beta hairpin and an alpha helix (beta/beta/alpha structure), and are the most common DNA-binding motifs found in eukaryotic transcription factors and also in prokaryotes. There were 214 C2H2type zinc finger genes identified in the silkworm genome, and this was the largest family of silkworm transcription factors (Figure 1C, Table 4). A previous report by Duan et al. (2008) identified the silkworm C2H2 family gene; most of the silkworm members belonged to evolutionarily conserved families, with the exception of some silkworm species-specific C2H2 genes. The C3HC4-type zinc-finger (RING finger) is a cysteine-rich domain of 40–60 residues that coordinates two zinc ions. Some proteins with this domain show activities as transcription mediators, coregulators, or suppressors. In total there were 51 C3HC4-type zinc-fingers identified in the silkworm. PHD-finger folds into an interleaved type of zinc-finger, chelating two zinc ions and thought to be involved in chromatin-mediated transcriptional regulation. For this family, a total of 39 genes were identified. In nearly all cases, zinc finger C4-type (two domains) is the DNA binding domain of a nuclear hormone receptor and involved in widely diverse physiological functions, including control of embryonic development, cell differentiation, and homeostasis (Moehren et al. 2004). In total, 13 genes of this family were identified in the silkworm genome. Zinc knuckle represents the CysCysHisCys (CCHC)-type zinc finger domains; some proteins with this domain show function as transcription factors. In total, 20 genes of this family were identified in the silkworm. GATA zinc finger uses four cysteine residues to coordinate a zinc ion and can bind to DNA; four genes of this family were identified in silkworm (Figure 1C, Table 4).

The homeobox (its name derived from its original identification in *Drosophila* homeotic) is a sequence that codes for a domain of 60 amino acids present in proteins of many or possibly all eukaryotes and binds DNA through a helix-turn-helix (HTH) structure. The homeodomain is found in many genes concerned with developmental regulation such as pattern formation, segmentation, cell-cycle regulation, and differentiation (Bondos and Tan 2001). This domain was the second largest family identified in the silkworm genome, with 78 genes (Figure 1C, Table 4). Chai et al. (2008) reported the identification of this family in silkworm, proposing that a subfamily *Bmshx* showed a lineage-specific expansion in silkworm compared to other insects.

Two common features in DNA-binding proteins are the presence of helical regions that bind DNA and the ability of the protein to dimerise. Both features are represented in the group of HLH proteins that share a common type of sequence motif. bHLH proteins are a group of eukaryotic transcription factors that exert a determinative influence on a variety of developmental pathways (Kageyama 1995). bHLH transcription factors were also a large family in the silkworm genome with a total of 35 genes (Figure 1C, Table 4). Other studies identified genes of this family in silkworm, and phylogenetic analyses revealed they were distributed in many bHLH subfamilies (Wang et al. 2007; Zhou et al. 2008).

The myb/SANT-like domain in Adf-1 (MADF_DNA_bdg) is an approximately 80amino-acid module that directs sequencespecific DNA binding to a site consisting of multiple tri-nucleotide repeats (Bhaskar and Courey 2002). In *Drosophila*, some transcription factors containing this domain showed the basis of its interaction with the alcohol dehydrogenase promoter, both as an activator to bind DNA in a sequence-specific manner and a coactivator to stimulate synergistic activation by *Dorsal* and *Twist*, or required for maintenance of female germline stem cells as well as oocyte differentiation (Pan, Huang et al. 1991; Baylies and Bate 1996; Stathopoulos and Levine 2002). In silkworm, 37 genes with myb/SANT-like domain were identified (Figure 1C, Table 4).

Maf transcription factors contain a conserved basic region leucine zipper (bZIP) domain, which mediates their dimerisation and DNA-binding properties, and play different roles in the cell (Hurst 1994). Three types of bZIP transcription factors including of bZIP_1, bZIP_2, and bZIP_Maf were identified. In mice, bZIP factor *MafA* contributes to specific transcriptional activity through the insulin promoter (Kajihara et al. 2003). In the silkworm, six, six, and two genes were identified belonging to bZIP_1, bZIP_2, and bZIP_Maf subfamilies, respectively (Figure 1C, Table 4). Song et al. (2009) reported that the bZIP-containing gene *BmCREB* was involved in the process of diapause induced by environmental factors.

Compared to the large families shown above, the remaining families contained fewer members. For example, the ligand-binding domain of nuclear hormone receptor, which is involved in binding the hormone, had 18 genes in the silkworm (Tanenbaum et al. 1998). The fork head domain, with function of involvement in early developmental decisions of cell fates during embryogenesis and dorsoventral patterning, had 15 members in the silkworm (El-Hodiri et al. 2001). T-box family proteins are found in a wide range of animals, but not in other kingdoms such as pants, performing DNA-binding and transcriptional activation/repression roles; 11 of these genes were identified in the silkworm (Wilson and Conlon 2002). The Mov34/MPN/PAD-1 family is found in proteasome regulatory subunits, eukaryotic initiation factor 3 (eIF3) subunits and regulators of transcription factors (Aravind and Ponting 1998); 10 members were identified in the silkworm. The ETS domain is rich in positively-charged and aromatic residues, and binds to purine-rich segments of DNA; eight of these genes were identified in the silkworm (Wasylyk et al. 1998) (Figure 1C, Table 4).

In contrast to the families mentioned above, the remainder were relatively small with < 10 members, including the Rel homology domain (RHD), P53 DNA-binding domain, CCAAT-binding transcription factor, and STAT protein (DNA binding domain) (Figure 1C, Table 4). Although these families are very small, they are conserved in many species.

### Transcription factors across the insect kingdom

To characterize the entire complement of transcription factors encoded by the other four insect genomes of *D. melanogaster, An. gambiae, T. castaneum* and *A. mellifera*, the same list of identified domains and e-value criteria were used to query corresponding proteome-predicted domains. The results showed that transcription factors accounted for about 4–7% of the total number of genes in insect genomes, similar to that of the model plant *Arabidopsis* (Riechmann et al. 2000) (Figure 1A). Comparing transcription factor content in genomes, silkworms showed a lower proportion (666 genes, 4.56%) than other insect species. In the model insect *D. melanogaster*, a total of 762 transcription factors were identified in this research, accounting for 5.56% of its genome. In the disease vector *An. gambiae*, a total of 658 transcription factors accounting for 5.06% of its genome were identified. In the pest *T. castaneum*, more genes were determined (total 811). However, the honeybee *A. mellifera* only had 665 transcription factors representing 6.58% of its genes and thus the highest proportion among the investigated insects.

### Basal transcription factors across the insect kingdom

The basal transcription factors were not as conserved across the insect kingdom as expected. Of the five insects, silkworm had fewest (36 genes) (Figure 1B), and *D. melanogaster* had the most (52 genes). Tables 5–8 list the gene ID, accession, e-value, protein ID, and description of identified basal transcription factors in *D. melanogaster, An. gambiae, T. castaneum*, and *A. mellifera*, respectively. Most of the basal transcription factor domains were identified in all insects, whereas some others were not conserved to the same degree. For example, the TFIIE alpha subunit and TAFII55 protein conserved region were not identified in the silkworm but had one or two members in other insects, indicating they were not very conserved in the silkworm. In other instances, transcription factor TFIIH complex subunit Tfb5 was only identified in *D. melanogaster*, NER and RNAPII transcription protein n terminal and RNA polymerase I-specific transcription initiation factor Rr were only identified in *T. castaneum*, and sigma-70 region 2 and sigma70 region 4 were only identified in *A. mellifera*.

### Other transcription factors across the insect kingdom

When searching the 230 other identified transcription factor domains, only 96 were determined in insects, illustrating that these other factor domains were not very conserved in all orders (Figure 1C, Table 2). However, the other transcription factor families among insects were rather conserved, since 76% (72 of 96 domains) were found in all five investigated insects. When comparing the other transcription factors silkworm with the other four insects, three features stood out.

First, most families were conserved in silkworm and the other four insects (Figure 1C). This was well demonstrated by large families such as the C2H2-type zinc finger, homeobox domain, C3HC4-type zinc finger (RING finger), HLH DNA-binding domain, PHD-finger, C4-type zinc finger (two domains), fork head domain, and ligand-binding domain of nuclear hormone receptor; they occupied the same important position in each insect. For example, the C2H2-type zinc finger family was about one-third of all transcription factors in each insect, and the homeobox domain was the second largest family in each insect. Most of the small families also illustrated the conserved feature among insects with similar numbers in each species, including Runt domain, bZIP Maf transcription factor, CP2 transcription factor, transcription factor DP, and transcription factor subunit Med 10 of Mediator complex. Tables 9–12 list the gene ID, domain accession, e-value, and domain ID of the identified other transcription factors of *D. melanogaster, An. gambiae, T. castaneum*, and *A. mellifera*, respectively.

**Figure 2.**
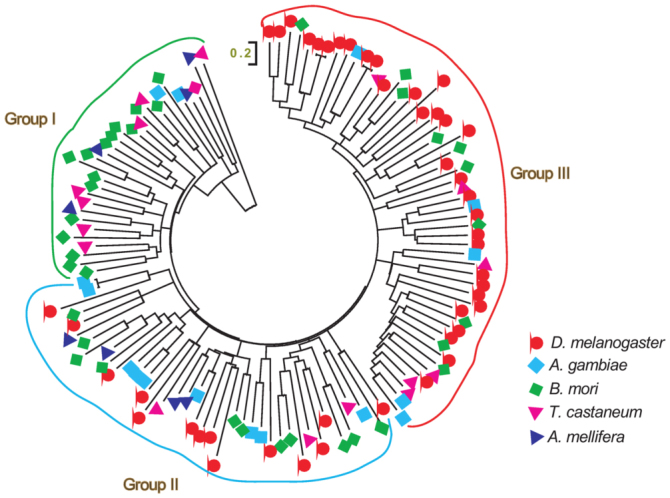
NJ phylogenetic tree of myb/SANT-like domain of insects *Drosophila melanogaster, Anopheles gambiae, Bombyx mori, Tribolium castaneum*, and *Apis mellifera*. High quality figures are available online.

Second, there were diverse gene numbers of most large families among insects (Figure 1C). This may be related to species-specific gene expansion in some families. For example, in the largest family C2H2-type zinc finger, 55.6% of the silkworm genes were species-specific; in *Drosophila*, the proportion was lower at 43.9% (Duan et al. 2008). In another example, in the second largest family homeobox domain, a new group of 12 genes (*Bmshx*) in silkworm had a relatively independent cluster relative to other insects according to phylogenetic analysis, indicating that *shx* subfamily was species-specific in silkworm (Chai et al. 2008). However, some species expansion genes were not clustered together as clearly as *Bmshx*. They showed a rather scattered pattern that did not indicate a species-specific gene expansion phenomenon. This could be typically seen in an alcohol dehydrogenase transcription factor Myb/SANT-like family (Figure 2). Although the silkworm Myb/SANT-like family genes mainly clustered in cluster I, there were still some genes of other insects in this cluster. Similarly, cluster III mainly contained *Drosophila* members, although there were several silkworm and other insect genes in this cluster. This may be caused by small gene duplication events in silkworm and *Drosophila*. For this family, cluster II contained genes of five insects with similar members.

Third, some very small families were not identified in all of the five insects. Overall, for all of the 96 insect domains, there were 14, 20, 18, 4, and 11 that were not identified in all of *D. melanogaster, An. gambiae, B. mori, T. castaneum*, and *A. mellifera*, respectivly. Among these domains, only the heat shock factor (HSF-type DNA-binding) domain was not identified in silkworm, but one or two members were found in the other insects. Heat shock factor is a transcriptional activator of heat shock genes and can bind specifically to heat shock promoter elements (Clos et al. 1990). The silkworm contained one HSF-type DNA-binding domain but its e-value was too low to be identified. Why this domain was not conserved in the silkworm is a question to be investigated. These non-conserved insect transcription factors may contribute to species-specific features.

### Expression patterns of *B. mori* transcription factors

***Expression profiles in multiple tissues*.** The silkworm genes oligonucleotide 70-mer microarray probes were designed using 6 times genome sequences (Xia et al. 2007).

Therefore, gene expression microarray was a good method to for determining expression profiles of the transcription factors at the genome level. Day three of the fifth instar was a very good period to investigate the transcription factors expression profiles of silkworm larvae (Xia et al. 2007); thus, using the silkworm multiple-tissue expression microarray data at this developmental stage, we investigated its transcription factor multiple-tissue expression profiles expecting to find some tissue-preference or tissue-universal transcription factors (Xia et al. 2007). In this analysis, a gene was considered to be expressed in a tissue if its signal intensity was > 400 signal-intensity units after subtracting the background (Xia et al. 2007). In total, 275 genes passed the filtering and illustrated expression during this developmental stage, including 23 basal and 252 other factors.

**Figure 3.**
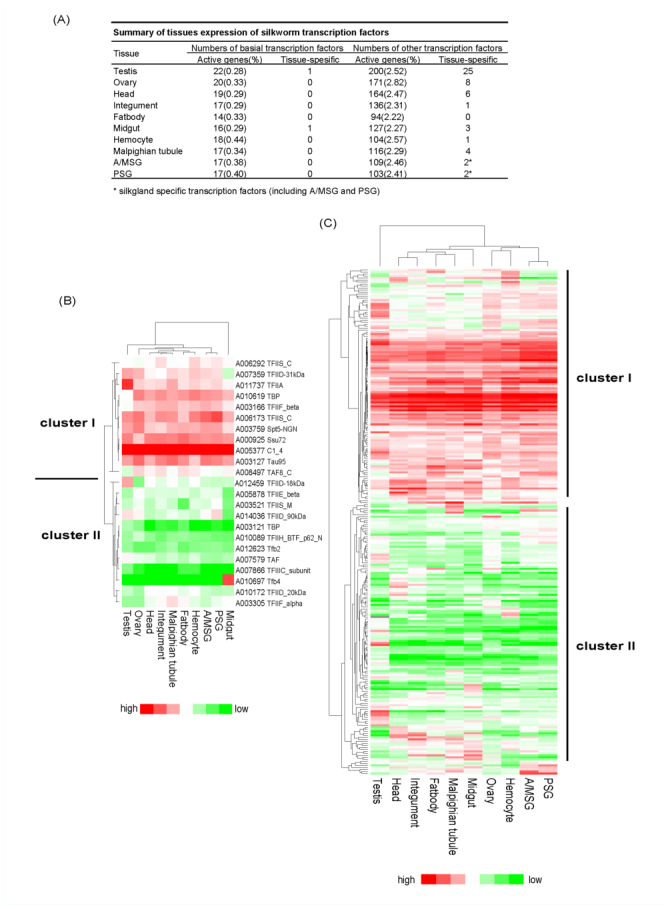
Multiple tissues expression on day three of the fifth instar of silkworm transcription factors. (a) Summary of tissues expression of silkworm transcription factors. Table 13 shows the tissues expression gene numbers of each family. (b) Hierarchical clustering of silkworm basal factors. We used the letter A to substitute letters BGIBMGA to simplify the expression of gene serial number (the same below). The list of basal factors microarray data are shown in Table 14. (c) Hierarchical clustering of silkworm other factors. The list of other factors microarray data is shown in Table 15. High quality figures are available online.

About 76.7% (23 of 30 with probes) of basal factors showed expression with similar numbers in each tissue, which accounted for about 0.3–0.4% of the tissues active genes in the tissues (Figure 3A). Hierarchical cluster analysis of multiple-tissue expression data showed two different expression patterns (Figure 3B). Cluster I represented genes with high expression signals in most tissues, including of C1_4 (A005377), Ssu72 (A000925), Tau95 (A003127), and TBP (A010619). For example, *btf* transcription factor (C1_4, A005377) had > 5000 signal units in every tissue, strongly illustrating its general expression feature. In contrast to cluster I, cluster II mainly represented genes with low expression signals in most tissues, such as TBP (A003121), TFIIIC_subunit (007866), and Tfb2 (A012623). Only A012623 (Tfb2) and A010697 (Tfb4) were identified as tissue-specific basal factors, with expression only in testis or middle gut. Table 13 shows the gene numbers of each basal domain in each tissue, and Table 14 shows the multiple-tissue expression microarray data of the basal factors.

Compared to the proportion of active basal factors in all basal factors (76.7%), there were lower proportions of the active other factors (44.9%, 252 of 561 with probes), clearly suggesting that other factors were not as generally expressed as basal factors. Although different numbers of active genes were found in each tissue, the proportions of active other factors of all active genes were very similar (about 2.5%) (Figure 3B). In the large families, most showed multiple-tissue expression with no significant tissue preference (Table 15). The expression of other transcription factors was also divided into two clusters (Figure 3C). Cluster I contained genes highly expressed in most tissues and, in contrast, cluster II contained genes seldom expressed in most tissues. Table 13 shows the gene numbers of other domains in each tissue and Table 15 shows their multiple-tissue expression microarray data. Each tissue could be identified with other tissue-specific factors with the exception of the fat body (Figure 3A). Among the 10 tissues, the testis had 25 testis-specific genes, which was much more than in other tissues. Why the silkworm testis expressed many more tissue-specific transcription factors and active genes is unknown; however, it can be speculated that this may be related to the development and function of the testis. There were less ovary-specific genes (eight genes), including families of zf-C2H2, bZIP_Maf, and Runt. Ovary-specific genes may be related to the function of the ovary and embryos. For example, the *runt* gene is located in the X chromosome and is responsible for the control of both sex determination and dosage compensation in *Drosophila* (Duffy and Gergen 1991; Torres and Sanchez 1992). In the sea urchin, *SpRunt-1* was a positive regulator of the aboral ectoderm-specific *CyIIIA* gene in embryos (Coffman et al. 1996). In silkworm, *Bmrunt* showed expression during embryonic development and is likely a vital regulator of the ovary (Liu et al. 2008). For highly specific silk-gland organ of the silkworm, only two tissue-specific transcription factors were identified (A005127 and A007303 of HLH family). A007303 showed higher expression in the posterior silk gland (PSG) than the anterior/median silk gland (A/MSG), and to this point their functions are unknown. However, previous research showed that the HLH domain was related to silk gland development in *Bombyx* (Matsunami et al. 1999). In the black widow spider, a HLH transcription factor was identified in the silk gland (Kohler et al. 2005). Overall, this analysis provided some clues for future research on silkworm organ specificity. Table 16 shows the multiple-tissue expression microarray data of the tissue-specific other factors.

***Expression profiles in spinning larvae, pupae and moths*.** During the silkworm life cycle from larval to adult stage, its phenotype and habit changes. Investigating the expression profiles of transcription factors from larvae, pupae, and adult would be useful for understanding its development and metamorphosis. Thus, samples of the whole body of five developmental stages including day three of the fifth instar larva, mature larva, spinning larva, pupa, and adult (moth) stages were used.

**Figure 4.**
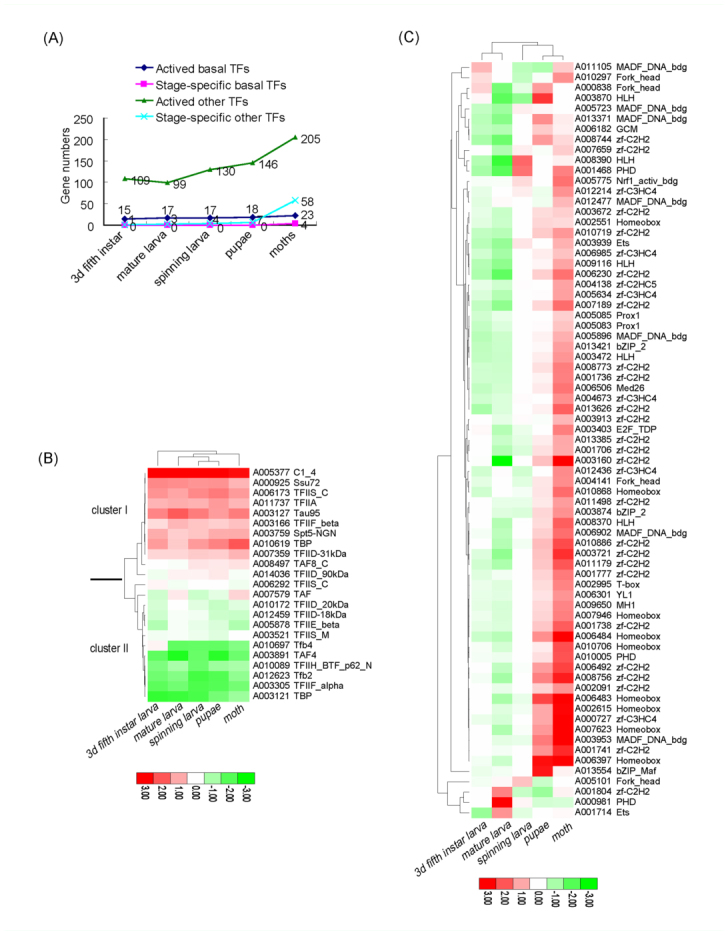
Expression of developmental stages of silkworm transcription factors. (a) Active transcription factor numbers of five developmental stages. (b) Hierarchical clustering of silkworm basal factors. (c) Hierarchical clustering of silkworm developmental stagespecific other factors. The list of microarray data of basal factors and developmental stage-specific other factors are shown in Table 17. High quality figures are available online.

The expressed numbers of silkworm transcription factors gradually increased from larva to moth (Figure 4A). For example, there were 15, 17, 17, 18, and 23 basal factors that showed expression at day three of the fifth instar, mature larva, spinning larva, pupa, and moth stages, respectively; of the other factors, there were correspondingly 109, 99, 130, 145, and 205 genes that showed expression. These results demonstrated that from larva to moth, the transcription of silkworm genes increased, and more specifically from pupa to moth.

The same tendency was found when investigating the stage-specific factors. Four basal moth-specific basal factors were identified, whereas none were found in other stages. For other factors, there were 1, 3, 4, 7, and 58 stage-specific genes identified at day three of the fifth instar, mature larva, spinning larva, pupa, and moth stages, respectively, showing a sharp increase at the moth stage (Figure 4A).

Based on hierarchical clustering, the active basal factors were clearly separated into high expression (cluster I) and low expression (cluster II) clusters in most stages (Figure 4B). Among cluster I genes, TFIIH C1-like domain containing gene A005377 showed > 6000 signal units during all five stages, again illustrating this general factor's characteristics during silkworm development. Compared to cluster I, cluster II genes showed lower expression signal units, included the four moth-specific genes Tfb2 (A012623), TAF4 (A003891), TFIIF_alpha (A003305), and TBP (A003121). Table 17 shows the microarray data of basal factors among the five developmental stages.

Similar expression profiles were found for other factors; many showed higher expression at all stages and the remainder showed lower expression. Among them, 17 genes showed high expression with signal units > 2000 at all five stages, indicating their universal expression feature during silkworm development. Stage-specific transcription factors might play important roles for corresponding developmental stages. Hierarchical clustering of the other stagespecific factors also clearly showed many more genes were expressed during the moth stage (Figure 4C). However, all of these stagespecific genes showed relatively low abundance of expression (< 1000 signal units), with the exception of A007623 (homeobox) and A003953 (MADF_DNA_bdg), which were highly expressed during the moth stage. Table 17 shows the microarray data of other factors among the five stages.

**Figure 5.**
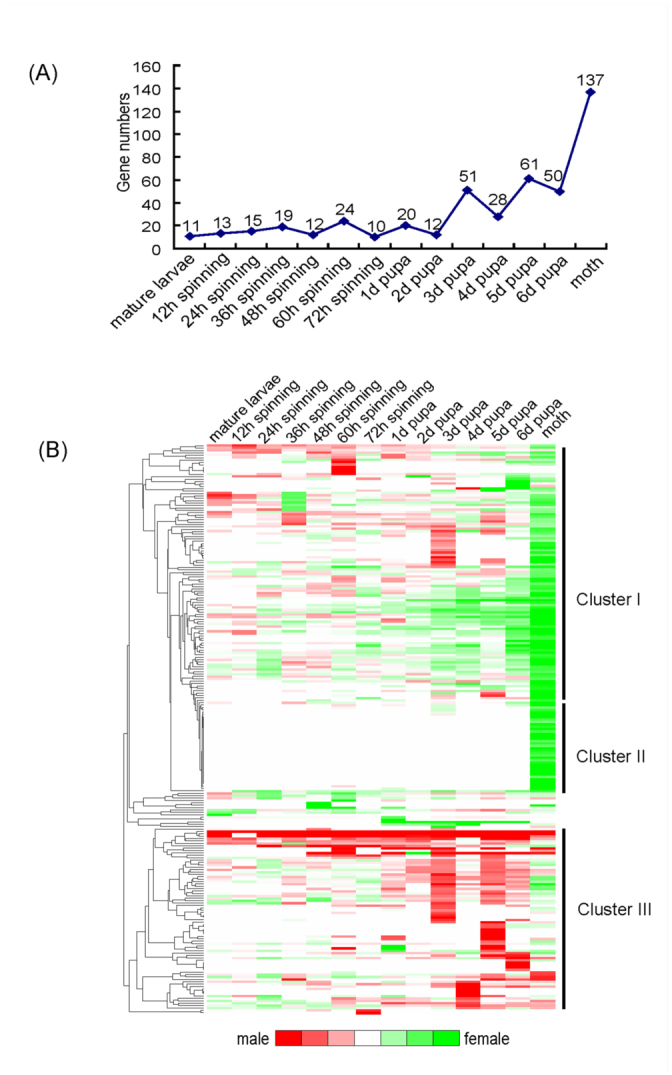
The expression of silkworm transcription factors between male and female. (a) Numbers of sexual differential expression genes during mature larvae to moth. (b) Hierarchical clustering of ratios of male to female during mature larvae to moth. The ratios of silkworm transcription factors are shown in Table 18. High quality figures are available online.

***Sexual dimorphism expression profiles*.** Expression of sexually dimorphic genes can result in sexual dimorphism, the most common biological phenomenon in nature. The silkworm is a female-heterogametic organism (ZZ in male, ZW in female) (Kovalevskaia and Filippovich Iu 1970). Hence, surveying the differences in sexually dimorphic transcription factors between sexes is of great interest. In total, 239 genes showed differential expression between sexes for at least one time point during the 14 time points of the four developmental stages investigated (Figure 5A). The number of differentialexpression genes increased from mature larva to adult. However, from mature larva to different time points of spinning larva, the numbers remained stable at about 10–20 genes. After that, from pupa of 1–6 days, the numbers increased up to about 60 genes. At the adult stage (moth), a total of 137 transcription factors showed differential expression. This kind of expression tendency was consistent with our previous analysis of expression profiles of all transcription factors. The results strongly suggested the disparity between male and female increased from mature larva to adult, supporting the increasing phenotype differences during this period of silkworm development.

Although there were many genes that showed stage-specific sexual differential expression, only one was identified as a sex-specific gene during all the investigated stages (male-specific gene, A010818, PHD). This gene was also a testis-specific high expression gene on day three of the fifth instar. Therefore, it could be used as a testis molecular marker, and we speculate that it has a vital role involved in the testis.

Hierarchical clustering of male-female ratios showed sexual differential expression profiles of the transcription factors (Figure 5B). Cluster I represented genes with a dynamic change profile between male and female during mature larva to moth, which gradually changed from higher expression in males at mature larva stage to higher expression in females at moth stage; this was especially evident at the moth stage, where almost all showed higher expression in females. Cluster II represented female higher expression genes at the moth stage. In contrast to cluster I, the expression abundance of most genes of cluster II increased gradually from mature larva to pupa stages in males. At the moth stage, about half showed higher expression in males, and the other half in showed higher expression in females. In general, most genes showed higher expression at pupa stage in males, and showed higher expression at moth stage in females. This result demonstrated that the expression character of sexually dimorphic factors was associated with developmental stage and more complex transcriptional regulation in females than males at moth stage, which might be related to the responsibility of spawning for the female moth. Table 18 shows the ratios of male to female of silkworm transcription factors at different developmental stages.

### Microbe-induced expression profiles of *B. mori* transcription factors

***Profiles induced by oral infection by microbes*.** While a pathogen is attacking, the host can activate transcription factors to trigger target genes' expression, caused by a series of signal recognition, modulation, and transduction. To understand the complex mechanism of host response to a pathogen, investigation of pathogen-induced transcription factors would be useful. Therefore, we used the silkworm's natural pathogens *B. mori* nucleopolyhedrovirus (BmNPV), Gram-positive bacteria *B. bombyseptieus*, fungal *B. bassinana*, and Gram-negative nonpathogenic bacteria *E. coli* to investigate this question. Most pathogens infect their hosts by contaminated food, thus oral infection was considered as close to natural infection and so was used in this analysis.

**Figure 6.**
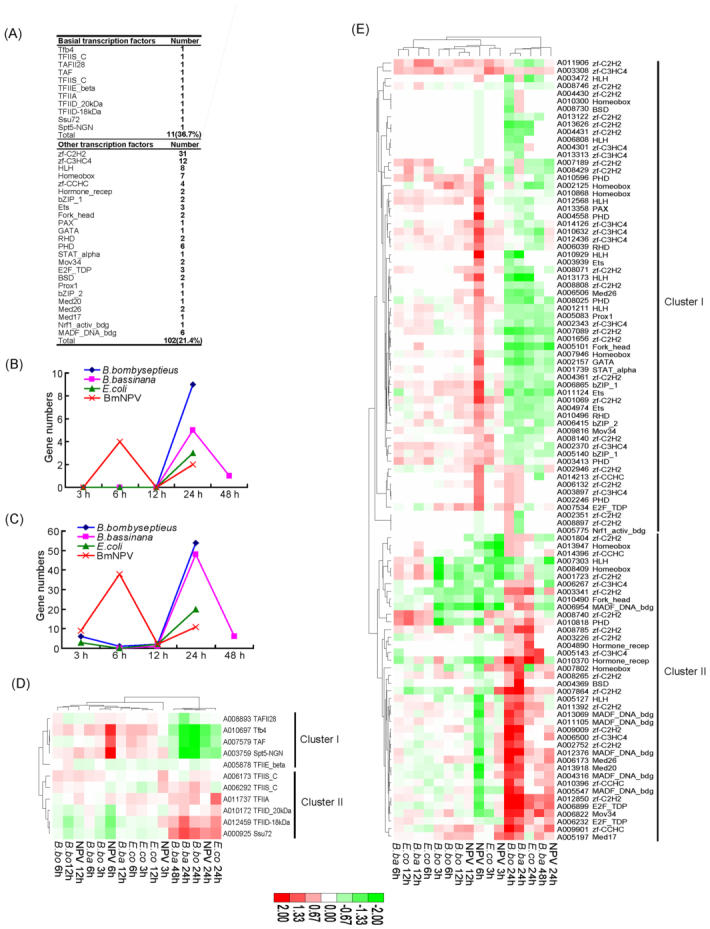
Analysis of the silkworm transcription factors response to *Bombyx mori* nucleopolyhedrovirus (BmNPV), *Bacillus bombyseptieus* (*B. bo*); *Beauveria bassinana* (*B. ba*) and *Escherichia coli* (*E. co*) oral infection. (a) Numbers of induced each family genes. (b) Induced basal factor numbers during the time course of infection. (c) Induced other factor numbers during the time course of infection. (d) Hierarchical clustering of ratios of microbes' induced basal factors. (e) Hierarchical clustering of ratios of microbes' induced other factors. The ratios of oral infection induced silkworm factors are shown in Table 19. The induced gene numbers of each domain are shown in Table 20. High quality figures are available online.

A total of 35 families were induced after infection, including 11 basal and 24 other families. Overall, there was a higher proportion of induced basal factors (36.7%, 11 genes) than of other factors (21.4%, 102 genes), suggesting that basal factors were more sensitive than other factors after oral infection by pathogens (Figure 6A). Of the induced factors, the largest family (the C2H2 finger type) showed 31 genes, and thus was the largest induced family. Unexpectedly, the second largest (the homeobox family) only showed seven genes, which was less than for the third (C3HC4 type or RING finger; 12 genes) and the fourth family (the HLH; eight genes), suggesting that the RING finger and HLH families were more sensitive than the homeobox family following oral infection by microbes. For the remaining families, less numbers were induced.

During 3–48 hours of the infection time-course of the four microbes, two induced profiles were clearly observed in basal and other factors (Figure 6B and C). One profile was a strong early response at six hours following BmNPV infection with four basal and 38 other factors. The other profile was a strong later response at 24 hours following *B. bombyseptieus, B. bassinana*, and *E. coli* infections. This result suggested a diverse pattern between the viral pathogen BmNPV and the other three microbes, which would be expected due to the different infection mode of BmNPV. Table 19 shows the induced ratios and Table 20 the induced numbers of each family by each microbe.

When comparing the four microbes, pathogens showed a stronger transcription factor response than the non-pathogen *E. coli*, thereby triggering a much stronger pathologic-physiological reaction. This was illustrated by the numbers of induced genes: 67, 59, 55, and 27 factors were induced by *B. bombyseptieus, B. bassinana*, BmNPV, and *E. coli*, respectively. Some of these genes were induced by four microbes, and some of the rest showed induction that was pathogen species-specific. Among the three pathogens, Gram-positive bacteria *B. bombyseptieus* (recently reported to cause the silkworm black chest septicaemia) induced more factors than others, possibly related to its high pathogenicity (Huang et al. 2009).

Hierarchical clustering analysis showed that the induced basal and other factors had similar expression profiles. Earlier infection (three, six, and 12 hours) and later infection stages (24 and 48 hours) were clearly separate (Figure 6D and E). Cluster I contained induced genes that were upregulated at the early stage of infection especially at six hours post-BmNPV infection, and downregulated later in infection (24 and 48 hours). Genes of this cluster might play vital roles in the host immune response. For example, RHD (Rel homology domain)-containing gene A010496 (*BmRel*) showed upregulation at six hours of BmNPV infection. Previous studies on *BmRel* showed an important role for the activation of antibacterial peptides of the Toll pathway (Kaneko et al. 2007; Tanaka et al. 2005). Thus, we speculate that the *BmRel* transcription factor in the Toll pathway plays a role following BmNPV infection, providing a clue to the mechanism of virus-mediated host immune response. In contrast, genes of cluster II were mainly downregulated during 3–12 hours, and upregulated during 24–48 hours, especially at 24 hours following *B. bombyseptieus* and *B. bassinana* infection. This cluster included many families such as zinc finger, MADF_DNA_bdg, Mov34, and Ets. They may play important roles in the regulation of pathogen-induced pathophysiological reactions.

Overall, our results demonstrated two visible features of the silkworm transcription factors after oral infection by microbes. First, BmNPV showed a very different induced expression pattern from the others. Second, transcription factors showed time-dependent dynamic expression profiles following infection by microbes. The most sensitive time point for BmNPV infection was six hours post infection, and 24 hours post infection was a key point for infection by the other three microbes. These data provide vital clues to understanding of the regulation of host response following oral infection by microbes.

**Figure 7.**
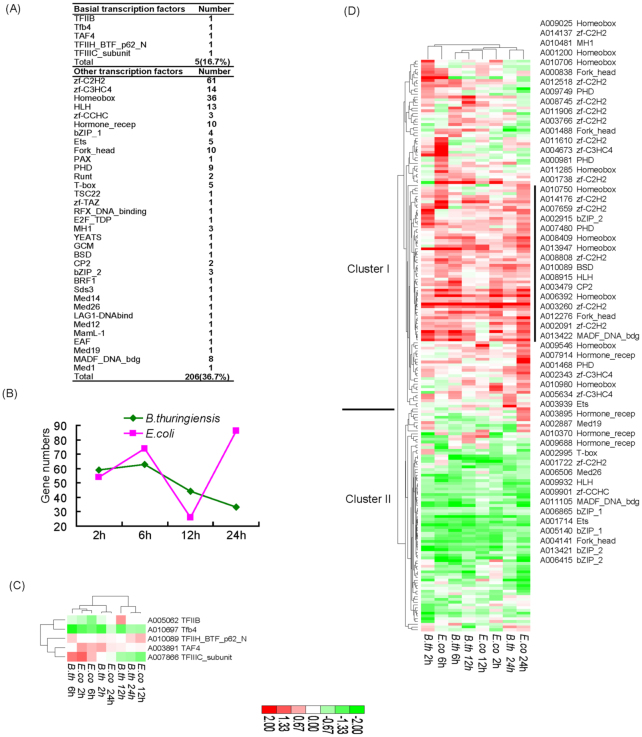
Analysis of the silkworm transcription factors response to *Escherichia coli* and *Bacillus thuringiensis* directly injection. (a) Numbers of induced each domain family genes. (b) Induced gene numbers during the time course of infection. (c) Hierarchical clustering of ratios of bacterial injection induced basal factors. (d) Hierarchical clustering of ratios of microbes' induced other factors. The right showed the gene information of vertical line included up-regulation factors during the infection. The ratios of injection induced silkworm factors are shown in Table 21. The induced gene numbers of each domain are shown in Table 22. High quality figures are available online.

***Bacterial injection-induced profiles*.** Besides natural infection, direct injection of bacteria into the insect body cavity is another common way to investigate modulation of host transcriptome and immune response. Moreover, previous reports have shown that there is a large difference between natural and directly injected infections (Loria and Padgett 1992). To further investigate this question, we analyzed microarray data of sampled fat-bodies after *E. coli* and *B. thuringiensis* direct injection. As expected, compared to oral infection (Figure 6), there were different expression profiles (Figure 7).

First, bacteria direct injection induced a much stronger host transcriptional response than oral infection, clearly illustrated by the two methods of *E. coli* infection. During 2–24 hours of the time course, a total of 161 transcription factors showed modulation by injection, whereas only 27 were identified following oral infection. A similar situation occurred for the two *Bacillus* spp. For *B. thuringiensis* injection, 136 genes showed modulation, which was more than twice that of *B. bombyseptieus* oral infection (59 genes). Second, after *E. coli* infection, the induced profiles of oral infection and injection were different (Figures 7B, 6B, 6C). Following injection, there was a small peak of induced numbers at six hours, whereas this time point showed a minimum for oral infection, demonstrating that direct injection could induce an early strong response in the host. This was also illustrated by *B. thuringiensis* injection with most genes induced at six hours. Third, the basal factors of injection showed less induced sensitivity than oral infection (five genes, 16.7%), whereas the other factors showed higher induced activity than oral infection (206 genes, 36.7%) (Figure 7A). Among injection-induced genes, the zfC2H2 and homeobox families were sensitively induced with 61 and 36 members, respectively. However, the homeobox family only showed seven genes induced by oral infection (Figure 6). The injection-induced bias was also observed in the hormonerecep family, with 10 members by injection and two by oral infection.

Among the two injected bacteria, different numbers of regulated genes were identified. This was mainly manifest at 12 and 24 hours post-injection (Figure 7B). At 12 hours, *E. coli* showed the least number of modulated genes (26), but with 44 genes this was not the least for *B. thuringiensis*. In contrast, *E. coli* showed the most modulated genes (86) at 24 hours, whereas *B. thuringiensis* showed its least genes during the 2, 6, 12, and 24 hour four time points (33). This may be associated with the different host immune responses to the two bacteria.

Cluster analysis of five injection-induced basal factors, showed that TFIIB (A005062) and TFIIIC_subunit (A007866) had dynamic expression profiles during infection (Figure 7C). Other factors showed similar expression profiles during the time course of *E. coli* and *B. thuringiensis* infection (Figure 7D). Cluster I mainly showed upregulated genes and Cluster II mainly showed downregulated genes by the two bacterial infections. In addition, more genes showed upregulation than downregulation during infection by the two bacteria. For example, many of homeobox, zf-C2H2, forkhead, PHD, bZIP_2, hormone_recep, and Ets members showed upregulation during infection, suggesting that these families were very active after bacterial injection. Table 21 shows the induced ratios and Table 22 the numbers of each domain of basal factors and other factors during *E. coli* and *B. thuringiensis* injected infections.

## Discussion

To the best of our knowledge, this is the first comprehensive presentation of transcription factors in *B. mori*. Previously reported TFs in insects including the bHLH family (Wang et al. 2007), the C2H2 family (Duan et al. 2009, the Homeobox family in the silkworm (Chai et al. 2008) and *T. castaneum* (Richards et al. 2008). Results of our study presented here used a more conserved expected threshold of < e-5 (compared to < e-1 or < e-10 previously used) and that resulted in lower numbers (Chen et al. 2008; Chai et al. 2008; Richards et al. 2008) However, the tendencies of the proportions of TFs accounted for all genes in each of these insects are similar. In the present study, results showed that transcription factors accounted for about 4–7% of the insect genome, which was similar to that of *Arabidopsis* (5.9%) (Riechmann et al. 2000). In the silkworm genome, the transcription factor component was 4.6%, which was lower than for other insects, possibly related to its relatively simple living environment. In contrast, the honeybee *A. mellifera* showed the highest component (6.6%), which may be related to its complex social behavior (Slessor et al. 2005). With the notable exception of *D. melanogaster*, this was the first survey of transcription factors components in model insects *An. gambiae, T. castaneum*, and *A. mellifera*. Compared to previous research on *D. melanogaster* (Riechmann et al. 2000; Jay 2006), our investigation separated basal and other factors and gave more detailed information about the domain families (Parrish et al. 2006; Riechmann et al. 2000).

Silkworm transcription factor components showed both commonalities and differences to that of the model plant *Arabidopsis* (Riechmann et al. 2000). For example, the C2H2-type zinc finger was the largest family for both species with > 200 genes, clearly showing their commonalities. At the same time, about 45% of *Arabidopsis* factors were from families specific to plants, suggesting a relatively large difference in gene transcriptional regulation between plants and insects. For example, Dof, WRKY, NAC, GARP etc., can only be identified in *Arabidopsis*, which were found to regulate some plant-specific functions such as photosynthesis or GA synthesis (Riechmann et al. 2000). Although the accurate function of most of these genes in the silkworm is unknown, it can be speculated that they play important regulated functions during the silkworm growth, development, differentiation, immune response, etc.

Unexpectedly, a rather conservative feature was found when comparing transcription factor families among insects. Most families were identified among the five investigated insects in similar numbers, revealing the conservative evolution of transcriptional regulation among insects. These insects follow similar life cycles but different life habits. For example, the silkworm produces silk and the bee gathers nectar honey. Thus, it can be speculated that the habits of insects are gradually formed through evolution and habit differentiation cannot presently be seen to a large degree at the gene level. On the other hand, functions associated with small amino acid changes could not be shown in this study. For example, the differences in spinning and flight habits between the domesticated silkworm and the wild silkworm were only suggested by SNPs (Xia et al. 2009). However, our report gives clues for exploration of the mechanism of transcriptional regulation among insects.

Expression profiles of transcription factors could help us understand potential regulation mechanism. In the silkworm, we found that at the developmental stage of day three of the fifth instar, active basal factors accounted for about 0.3–0.4% (23 genes) of the active genes in each tissue. For other factors, the proportion was 2.5% (252 genes). Of the 10 silkworm tissues, the testis showed the greatest number of factors expressed, which was positively correlated with the numbers of all genes activated in testis and testis-specific genes, indicating complex regulation of testis gene expression. Unexpectedly, the highly specific silk-gland organ of the silkworm only showed expression of two specific factors (A005127 and A007303 of the HLH family).

During development from larva to adult, the silkworm transcription factors expression showed a large change at the moth stage. Many more factors showed expression and many were identified as stage-specific genes at the moth stage. At the spinning stage, the factors showed stable expression, suggesting simpler transcriptional regulation mechanism of spinning than metamorphosis. Sexual dimorphic factors also increased from day three of the fifth instar to moth stage, which was connected with the gradually increased individual differences between male and female. Overall, these data leave the intriguing question open as to how these transcription factors are implicated in regulating the fate of protein-encoding genes during their consequent contribution to tissue differentiation and organogenesis, development, and sexual dimorphism.

Expression profiles of transcription factors induced by pathogenic microbes would be helpful to reveal the regulation mechanism of immune response and molecular pathology. After oral infection, the silkworm transcription factors showed the strongest early response (6 hours) to BmNPV. This was consistent with previous reports, showing that BmNPV can enter the host cell nucleus at an early stage of infection (6 hours) and thereby induce a strong host response (Ge et al. 2009; Tian et al. 2009). However, in contrast to BmNPV, *B. bombyseptieus, B. bassinana*, and *E. coli* mainly induced a strong later response (24 hours), suggesting a very diverse expression pattern between BmNPV and the other three microbes. Direct injection of bacteria into the silkworm body cavity induced a stronger response in transcription factors compared to oral infection, consistent with the biological phenomenon that direct injection of the same dose of bacteria can kill the silkworm much faster than oral infection. Of the regulated genes, only four were induced by both oral infection and injection of *E. coli*, including of A010370 (Hormonerecep), A010818 (PHD), and A013947, and A002125 (Homeobox). The results suggested that there was a large difference in transcriptional regulation of host gene expression between oral infection and injection. Our report is the first investigation of transcription factor response to pathogens and provides rich information for further understanding the relationship between pathogens and the silkworm host.

In the present report, in total 666 transcription factors including 36 basal and 630 other factors were identified in the *B. mori* genome. Totals of 762, 658, 811, and 665 transcription factors were also identified in *D. melanogaster, An. gambiae, T. castaneum*, and *A. mellifera*, respectively. The silkworm shared rather conserved transcription factor families among the insect kingdom. There were 275 silkworm factors with multiple-tissue expression on day three of the fifth instar, including 23 basal and 252 other factors. During the developmental stages of day three of the fifth instar, mature larva, spinning larva, pupa, and moth, many more genes were expressed at the adult stage. From mature larva to adult, the numbers of sexually differentially expressed factors increased. Pathogens BmNPV, *B. bombyseptieus*, and *B. bassinana* induced more factors than nonpathogen *E. coli* following oral infection. Bacterial direct-injection induced a stronger silkworm host transcriptional response than oral infection. Our investigation provided vital clues to understanding the regulation of silkworm development and responses to pathogen infection.

## Author contributions

LLH and QYX developed and designed the experiments. LLH, PZX, TCC, and TF performed the experiments. LLH analyzed the data and wrote the manuscript.
